# Registered Nurses’ experiences of caring for persons with dementia expressing their sexuality

**DOI:** 10.1002/nop2.1197

**Published:** 2022-02-15

**Authors:** Mari‐Louise Nilsson, Magdalena Annersten Gershater, Mariette Bengtsson

**Affiliations:** ^1^ Department of Care Science Faculty of Health and Society Malmö University Malmö Sweden

**Keywords:** dementia, elder care, nursing homes, registered nurses, sexuality

## Abstract

**Background:**

Sexuality is an integral part of human beings and persons living with dementia still perceive negative attitudes from caregivers in this regard.

**Aim:**

This study aimed to explore registered nurses' experiences of caring for persons with dementia living in nursing homes and expressing their sexuality.

**Methods:**

A qualitative inductive design was adopted; data were collected through semi‐structured interviews and analysed through content analysis.

**Results:**

The analysis reflected three categories. (1) Dealing with different reactions and responding to nursing staff and relatives: The registered nurses experienced discomfort, insecurity, frustration, distress and embarrassment when confronted with sexual expressions in people with dementia. (2) Caring with a focus on the person: The registered nurses expressed the importance of protecting the integrity of the person and consequently their right to sexual expressions. (3) Needing more competence development: The registered nurses expressed the need to educate and inform not only themselves but also the nursing staff and relatives.

## 1 INTRODUCTION

Sexuality is an important part of life, and the expression of each and everyone's sexuality is a human right recognized in national and international human rights documents. Even so, there is no uniform definition of sexuality. There is, however, a working definition developed on behalf of the World Health Organization (WHO):

Sexuality is a central aspect of being human throughout life and encompasses sex, gender identities and roles, sexual orientation, eroticism, pleasure, intimacy and reproduction. Sexuality is experienced and expressed in thoughts, fantasies, desires, beliefs, attitudes, values, behaviors, practices, roles, and relationships. While sexuality can include all these dimensions, not all of them are always experienced or expressed. Sexuality is influenced by the interaction of biological, psychological, social, economic, political, cultural, ethical, legal, historical, religious and spiritual factors. (WHO, 2006 )

The definition gives the impression that sexuality is a broad multidimensional concept and contributes to a person's identity. The desire or need for sexual activity does not disappear with age or cognitive impairment (Bauer et al., 2012; Casta‐Kaufteil, 2003), but old people and persons with a cognitive impairment often face prejudices and a form of stereotyped view of them as being asexual (Dominguez & Barbagallo, 2016). Every human has the right to sexual freedom and, accordingly, to make their own decisions about their sexual needs and desire (World Association for Sexual Health, 2017). To fulfil them is important for their health, well‐being and quality of life (Benbow & Beeston, 2012; Lindau & Gavrilova, 2010). Nevertheless, problems can arise in relation to sexual expression and behaviour, and it can be challenging for Registered Nurses (RNs) working in nursing homes to handle how old persons express their sexuality (Thys et al., 2019).

## 2 BACKGROUND

Dementia is an umbrella term for several diseases that are principal contributors to cognitive impairment and the need for care. According to figures from WHO (2015), the number of people living with dementia was estimated at nearly 47.5 million worldwide in 2015, a number that is predicted to increase to 75.6 million in 2030. In Sweden, 130,000–150,000 people currently live with dementia, and each year 20,000–25,000 persons develop the disease. Dementia is associated with a progressive, often irreversible loss of the ability to function independently in biological, social and occupational domains of life. More women are affected than men and a majority is living in nursing homes that give full‐time social and medical care (The Swedish National Board of Health and Welfare, 2017). At nursing homes, RNs are responsible for clinical decisions that offer persons with dementia opportunities to improve, maintain or regain their health; to manage health problems, illness or disability; and to achieve the best possible well‐being and quality of life until death. The nursing staff implement those decisions after a written delegation and may be involved in planning the necessary actions (Cooper et al., 2017). A person with dementia experiences physical and emotional changes over time because of their condition. The desire and longing for intimacy also varies from person to person and the progression of the disease can make it difficult to express sexuality in a proper manner. Persons with dementia still have their sex drive, but changes in their brains can make them act in ways that are new, different and sometimes inappropriate (Higgins et al., 2013). Persons with dementia living in nursing homes express their sexuality either verbally or physically. Sexual behaviour, such as public masturbation, hyperactive sexuality and attempts at unwanted intercourse with other patients, may occur in every stage of dementia (Alagiakrishnan et al., 2005). RNs face a wide range of experiences and emotions when confronted with expressions of sexuality and intimacy by persons with dementia. Nurses need to adopt a supportive approach when dealing with these highly sensitive situations (Thys et al., 2019). There is a tension between ensuring the autonomy of the person with dementia and their right to express their sexuality, and protecting family and other residents from harm (Wiskerke & Manthorp, 2019; D’cruz et al., 2020). Issues related to sexuality combined with consent and ethical demarcation can be complicated to handle and put a heavy burden on nursing staff and relatives (Mahieu et al., 2014). Caregivers’ own values influence how nursing actions are carried out (Gott, 2005; Doll, 2012; Villar et al., 2014; Simpson et al., 2017), and a previous study by Villar et al. (2018) has shown that the nursing staff often express anger and revulsion when encountering people with dementia expressing their sexuality. As sexuality is an integral part of human beings and persons living with dementia still perceive negative attitudes from caregivers in this regard, it is important to further explore this topic. Previous research has showed nursing staffs’ understandings of caring for persons with dementia expressing their sexuality but also RNs’ experiences need to be explored. RNs are essential to nursing actions in dementia care and, therefore, it is important to perform this study.

## 3 AIM

This study aimed to explore RNs’ experiences of caring for persons with dementia living in nursing homes and expressing their sexuality.

## 4 DESIGN AND METHOD

This study employed an inductive qualitative design based on semi‐structured interviews analysed with content analysis.

### 4.1 Study setting

The study was conducted in 2018. The participants in the study were ten RNs employed in eight different nursing homes situated in southern Sweden. The RNs worked during the day and were responsible for caring for 20–40 patients. One of these nursing homes only cared for persons with dementia, whilst the others cared for persons with a mix of diagnoses, including dementia. In each of these nursing homes, one to two RNs were in charge of nursing. In Sweden, the RNs are solely responsible for the nursing actions, although these are in most cases executed by assistant nurses/nursing staff. Each person living in nursing homes has their individual physician. Medical needs are conveyed to each physician by the RNs when the need is identified. Nursing homes give homes intended for full‐time living for people with dementia, together with interventions in the form of health and social care for elder people in need of special support (The Swedish National Board of Health and Welfare, 2017).

### 4.2 Sample and recruitment

The first author contacted nursing homes in southern Sweden and informed the RNs and the nursing home managers about the aim of the study. Those who expressed their interest were then included as participants. The intention of the study was presented in both oral and written form, and the RNs and the nursing home managers gave their written consent to participate in the interviews. Further, informed consent was obtained by making sure the RNs understood the given information about the aim and setup of the study, and the opportunity to terminate the participation at any given moment. A convenience sample was used (Polit & Beck, 2016). The age of the ten participating RNs ranged between 30 and 67 years (median = 55), and they had worked as RNs in various settings for between 7 and 37 years (median = 15). Out of the 10 participants, nine were women.

### 4.3 Data collection

The data were collected through semi‐structured interviews. Initially, a pilot interview was conducted to ensure the quality of the questions. The pilot interview was included in the results. The ten interviews revolved around the key question “What is your experience of caring for persons with dementia expressing their sexuality?”. They contained follow‐up questions, such as “How did you handle it?”, “What kind of emotions did it arouse in you?”, “Could you please develop that?”, “Do you have more examples of similar situations to this one?”. In connection to the interviews, a short summary of the conversation was carried out in collaboration with the informants, a so‐called member check (Burnard, 1991). The interviews were recorded and transcribed verbatim by the first author, who also performed the interviews. The interviews took place in the nursing homes where the RNs worked, and they lasted from 29 to 62 min (median = 43 min).

### 4.4 Data analysis

All authors participated in the analyses of the collected and transcribed data, discussed the relevance of the result and formed the categories and subcategories together. Content analysis was used to examine the transcribed text, as described by Bengtsson (2016) and the interpretation of the material were conducted on a manifest and a descriptive level. Four distinct stages of content analysis were used as follows: decontextualization, recontextualization, categorization and compilation (Bengtsson, 2016).

During the *decontextualization* process, all the authors familiarized themselves with the data and read the transcribed text to obtain a sense of the whole. Meaningful units were identified and coded, and a code list was established to secure reliability. The aim was to find a recurring pattern in the data, and this process was repeated several times. On each occasion, the process started at a new place in the text to further strengthen the study's trustworthiness. The *recontextualization* stage took place after the meaning units were identified. The authors checked that all aspects of the content were relevant to the objective of the study, and the material was coded.

**FIGURE 1:**
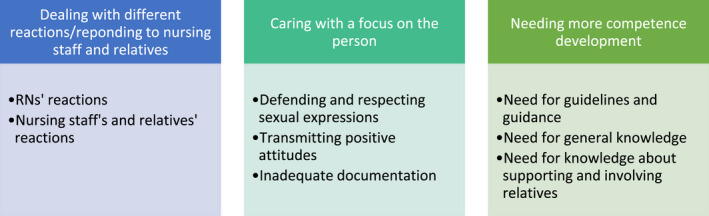
Registered Nurses’ experiences of caring for persons with dementia living in nursing homes and expressing their sexuality (results shown in three categories and subcategories)

The *categorization* stage involved creating categories for the data. To extract themes from the data, the authors divided the coded material into broad groups, based on the different intentions of the study. Quotes were included in the synthesis of the data to additionally strengthen the validity and credibility of the analysis (Bengtsson, 2016). The categorization ended, when a realistic elucidation was reached. In the *compilation* stage, the data were presented from as neutral a perspective as possible whilst also considering the objectivity of the data. For a quick overview of the summary of categories and subcategories, see Figure 1.

### 4.5 Ethical considerations

Sexuality can be a sensitive subject, and there was a risk of the RNs feeling embarrassed when talking about the subject. Further, the facts that identified in the interviews pertained to persons with dementia and their behaviour, which may have prompted ethical reflections amongst the RNs about whether it was right to share this information, as the persons had not consented. Therefore, no names were mentioned in the interviews, and the stories could not be traced to any single person. It was important to create an atmosphere of trust and confidence by listening and confirming during the conversation when there were situations that were difficult to talk about. Signed informed consent was obtained after the RNs had read the information about the study. The study follows the CODEX (2018) guidelines set by the Swedish Research Council. The Act concerning the Ethical Review of Research Involving Humans (SFS 2003:460) does not apply to this study, so Research Ethics Committee approval was not needed.

## 5 RESULTS

The result revealed that the RNs experienced a need to carry out person‐centred care, where defending and respecting the sexual expressions of each person was important. The RNs tried to transmit positive attitudes to the nursing staff to make them perform person‐centred care. It was also shown that inadequate documentation of sexuality was a hindrance to person‐centred care. Furthermore, the RNs expressed the troublesome experience of having to deal with and respond to the different reactions of the nursing staff and relatives and dealing with their own reactions. The interviews revealed that the RNs found it difficult to know whether they were doing the right thing in nursing situations, in providing guidance to staff, and in involving relatives about sexual expression in persons with dementia. Accordingly, the RNs expressed a need for reflection, education, competence and guidelines on the topic. The following categories identified from the RNs’ experiences: (1) Dealing with different reactions and responding to nursing staff and relatives, (2) Caring with a focus on the person and (3) Needing more competence development. Figure 1 presents the results more clearly.

### 5.1 Dealing with different reactions and responding to nursing staff and relatives

Caring for persons with dementia who express their sexuality induces reactions. The RNs had to deal with their own reactions whilst also dealing with reactions from nursing staff and relatives.

#### 5.1.1 RNs’ reactions

The RNs experienced discomfort, insecurity, frustration and embarrassment when they were confronted with persons expressing their sexuality. Furthermore, feelings of insecurity identified when they were uncertain about having acted “correctly” towards persons expressing their sexuality. The RNs also expressed sympathy for the person and their visiting relatives and experienced the situation as tragic and distressing for both parties when the person with dementia openly showed their sexuality.

*As an RN, you want to have a certain amount of control, but when it comes to sexual expressions, it’s totally worthless. Is it my own values that are in control? What kind of expressions is this? I feel frustrated*. (Interview 3)

#### 5.1.2 Nursing staff's and relatives’ reactions

The RNs had to deal with the nursing staff's reactions of distress, disgust and discomfort when persons with dementia expressed their sexuality. According to the RNs, younger nursing staff were more likely to feel uncomfortable when sexual expressions occurred. The RNs had to deal with it by letting more experienced staff members handle the persons expressing their sexuality. Moreover, the RNs sometimes had to handle the nursing staff's feelings of sympathy with the relatives, when the person with dementia met their relatives and expressed their sexuality in a way that could be perceived as undignified for everyone involved. Some relatives had a hard time understanding the person's sexual expression, and many felt distressed. Consequently, the RNs expressed a need to alleviate their distress.

*The wife had a hard time understanding that her husband had changed because of the disease. The man started to like a co‐patient and they hugged and kissed. His wife became upset. We talked a lot about this in the personnel group. Is this ethically correct? The wife took it badly and felt horrible*. (Interview 7)

### 5.2 Caring with a focus on the person

The interviews revealed that the RNs perceived person‐centred care as important for good quality in nursing. The RNs highlighted the importance of providing safe, good‐quality care. They also emphasized the well‐being of the person and that, in this, sexuality was an important aspect. The RNs explained that focusing on the persons’ needs and showing respect for those needs proved to be beneficial for the persons with dementia and were, thus, good nursing practices.

#### 5.2.1 Defending and respecting sexual expressions

According to the RNs, there was a need for and a will to protect the integrity of the person and, consequently, their right to sexual expressions. They considered performing person‐centred care an important part of safe care of good quality.

Further, the importance of respecting the person's sexual rights identified as a theme in the interviews. Although sexual expression is a human right, it often created a dilemma for nursing staff. When persons with dementia express their sexuality, they may not be able to see the consequences of their behaviour. For instance, sexual expressions such as masturbation were common amongst persons with dementia, and if this was done in public, it was important for the nursing staff to divert them and take them to a secluded place. The RNs expressed difficulties to, on the one hand, facilitate the right of the persons to express their sexuality and, on the other hand, protect them from the consequences. In some cases, the RNs felt the need to inform the nursing staff that the persons were entitled to their sexuality and that the nursing staff's attitudes and values should not affect the caring.

*Our values should not be in control. They have all the rights. There could be a lot of ethical opinions and thinking among the staff. Some of them think it’s unpleasant and weird. The RNs’ role in this is to balance. It’s all about care and [finding] a good way to take care of people so that they can feel good*. (Interview 10)

#### 5.2.2 Transmitting positive attitudes

The RNs experienced that it was useful to encourage positive attitudes in nursing staff, to not dramatize the topic of a person's expression of their sexuality, and to support the nursing staff when the person engaged in sexual behaviour. This was done by the RNs showing and highlighting situations when persons with dementia expressed their sexuality, and that in turn brought joy to the nursing staff caring for them. It was important for the RNs to emphasize positive attitudes towards sexual expression by communicating such situations, to encourage the nursing staff to adopt a similar outlook.

*There was a lady with dementia and a gentleman with dementia who both lived in the same care facility. They sat together in the sofa and held each other’s hands as they watched TV. He also went to her room. It was sweet. They were so in love with each other. Yes, it was cute*. (Interview 6)

#### 5.2.3 Inadequate documentation

Another important issue expressed by the RNs was the uncertainty about how to document sexuality; consequently, there was a lack of documentation in this regard. Although sexuality was included as an option in the documentation system, there was poor knowledge amongst staff about how and when to document sexual expressions. The RNs perceived lack of documentation as an obstacle in the nursing process and in person‐centred care.

*It* *is not often we document something about sexuality*. *It* *is not a topic that we address*. (Interview 2)

### 5.3 Needing more competence development

The RNs emphasized the need for more education and increased competence to guide, educate and inform not only themselves but also the nursing staff and relatives. They expressed their concern for the lack of guidelines and guidance in nursing concerning the topic.

#### 5.3.1 Need for guidelines and guidance

According to the RNs, the more the nursing staff talked about persons with dementia and expressions of their sexuality, the more they learned, and their shyness disappeared. The RNs also established that it was important to support the nursing staff and guide them in situations that arose.

The RNs stated that because the subject of persons with dementia and their expressions of sexuality was difficult, it required clear guidelines.

*There is a need for guidelines, as you feel insecure in certain decisions. If I start from the patient, it often feels good, but it would have been good to have guidelines so I can show the staff*. (Interview 5)

#### 5.3.2 Need for general knowledge

Another aspect of the RNs’ experience concerns general knowledge about the subject of sexuality and persons with dementia. The RNs requested tools to be able to supervise care staff and relatives, tools in the form of forums for discussion and reflection and educational materials and guidelines. Knowledge could give a sense of security in caring for the persons with dementia and a greater freedom in bringing up the subject of sexuality and formulating nursing interventions.

I would have liked the topic to be discussed in a group where you had some educational material, such as a case study. (Interview 1)

### 5.4 *Need* *for knowledge about supporting and involving relatives*

Supporting relatives and increasing their understanding of how expressions of sexuality could manifest in persons with dementia, was an important nursing intervention, according to the RNs. In addition, experiences identified showing that the RNs did not always dare to talk to relatives about the subject, because of too little knowledge and the sensitivity of the topic.

We as nurses are bad at discussing this I think you find it difficult. When I look back at the nurses I have meet, not many have given expression to discussing sexuality. We discuss side effects of drugs because it is easy, manageable, and measurable, but sexuality is just difficult. (Interview 8)

## 6 DISCUSSION

To the best of our knowledge, this study is the first one performed in Sweden that highlights RNs’ experiences of caring for persons with dementia who express their sexuality. Nursing care puts demands on nurses’ ability to deal with their own and others’ reactions, and the present study sheds light on this aspect as an important factor when dealing with persons with dementia and their sexual behaviour. This is supported by previous studies on the topic, which have shown that the prevailing view of persons with dementia expressing their sexuality is generally conservative and negative, and that it is difficult for RNs and nursing staff to master feelings of embarrassment, shame, insecurity and discomfort (Alagiakrishnan et al., 2005; Bronner, 2015; Mahieu et al., 2011).

RNs face a wide range of experiences and reactions when confronted with expressions of sexuality and intimacy by persons with dementia. They need a supportive approach to guide them in dealing with these highly sensitive situations (Thys et al., 2019).

The RNs in this study expressed the importance of protecting the integrity of the person with dementia and consequently their right to sexual expressions. As mentioned above, the RNs emphasized a need for the time and opportunity to discuss the issue in a forum where all the nursing staff had the opportunity to express their feelings and experiences. At the same time, such a forum would allow them to obtain advice and exchange knowledge. Studies show the need for RNs to have an open communication in the team caring for the person with dementia, where ethical dilemmas linked to persons with dementia and sexuality can be reflected upon (Alagiakrishnan et al., 2005; Saunamäki et al., 2010; Di Napoli et al., 2013; Bronner, 2015; Makimoto et al., 2015).

The RNs in the present study wanted more guidance in caring for persons with dementia who express sexuality. A previous study has concluded that clinical group supervision is one way to support RNs and nursing staff when it comes to handling the sometimes burdensome emotional part of nursing. Clinical supervision has been conducted since the 1980s, and it has been extensively evaluated. The results of the evaluations show that RNs, nursing staff and relatives can receive support in their emotional work and ethical dilemmas through these group meetings (Brunero & Stein‐Parbury, 2008).

Further, previous studies indicate that educational efforts concerning sexual expression in persons with dementia could be of great importance to RNs, nursing staff and relatives (Gott, 2005; Mahieu et al., 2011; Doll, 2012; Di Napoli et al., 2013; Villar et al., 2014; Simpson et al., 2017). The fact that RNs seek guidelines is to be expected; it is a need that naturally arises when care is complicated and different ethical principles collide. Several studies have highlighted the need for guidelines about persons with dementia expressing their sexuality (Ehrenfeld et al., 1997; Saunamäki et al., 2010; Mahieu et al., 2014; Makimoto et al., 2015; Simpson et al., 2017). However, the question is to what extent staff (and RNs, in this case) can be helpful in this complicated and often contradictory reality of nursing. Guidelines cannot cover the multifaceted and complicated care that often must be managed on a case‐by‐case basis.

Another important issue reported by the RNs was the uncertainty about how to document issues of sexuality and the consequent lack of documentation. If nursing diagnoses are not formulated properly, it is impossible to communicate nursing actions/interventions and consequently difficult for the nursing staff to carry out the necessary interventions. When the staff do not work uniformly according to the nursing actions prescribed by the nurse, it is not possible to evaluate their nursing practice and there is a risk of all nursing staff doing things their own way (Barmon et al., 2017). In this context, documentation according to the nursing process must be an absolute necessity, because each person's needs are unique and the RNs and the nursing staff must work out what actions need to be performed for each individual (Rösvik et al., 2011).

To ensure proper documentation and to formalize a continuous evaluation of situations involving sexual expression based on a person‐centred approach to nursing about persons with dementia living in nursing homes, we recommend the use of a quality register. The purpose of the quality register for Behavioral and Mental Symptoms in Dementia (BPSD) is to ensure the quality of care for people with dementia and to increase their quality of life. The register has a clear structure for evidence‐based nursing measures and an evaluation of proposed interventions (Swedish BPSD Register, 2020).

### 6.1 Limitations

The interviews were conducted with RNs in a limited context and geographical area, which may affect transferability.

Including only nursing homes, and excluding ordinary accommodation, is a limitation affecting both the representation of the population and the transferability of the results (Lincoln & Guba, 1985). Only RNs who worked during the day were included in the study and excluding RNs who worked at night may have affected the results, as sexual expressions are likely to occur also at night. Some nursing homes had two RNs and, due to the workload, only one RN could participate. A survey would have made it possible to increase the number of participants and give a fuller result, but the advantage of interviews is the flexibility they entail: Individual self‐reported feelings, attitudes and beliefs can be captured in a way that is impossible in a survey (Bennett et al., 2010).

The data were analysed through content analysis, as described by Bengtsson (2016), which gave an opportunity to structure and present the results according to themes. There is always a risk of subjectivity in data interpretation because it is possible to interpret the text in different ways. In order try to overcome this, all steps in the analysis process have been described, and the process of the content analysis was repeated on several occasions by all the authors to finally come to a consensus (Elo et al., 2014). The communicative validity of this study relates to the subject for discussion. In all the themes revealed in the study, the description has been as exhaustive as possible. However, the results are limited by the small sample size and the participants’ geographical location. The interviews in this study reflect the participants’ subjective retro‐perspective experiences and perceptions, not necessarily authentic facts, which could also have affected the results.

## 7 CONCLUSION

Caring for persons with dementia living in nursing homes and expressing their sexuality is perceived by RNs as hard and challenging. The lack of documentation is an obstacle in the nursing process; it creates a risk of not performing person‐centred care and delivering high‐quality nursing interventions. Being confronted with burdensome feelings and facing ethical dilemmas in the care for persons with dementia expressing their sexuality creates a need for team‐based reflection and support. Moreover, education is needed for anyone involved in this context. Future research should be focusing on evaluating nursing care for persons with dementia expressing their sexuality, when using the nursing process as intended, with a systematic guide to person‐centred care including assessment, diagnosis, planning, implementation and evaluation. As far as we know, there is limited research that explores the experiences of RNs in nursing homes caring for persons with dementia expressing their sexuality.

## CONFLICT OF INTEREST

There are no conflict of interest for Mari‐Louise Nilsson, Mariette Bengtsson and Magdalena Annersten Gershater.

## AUTHOR CONTRIBUTIONS

The first author carried out the design of the study and performed the interviews. All authors participated in the analyses of the collected and transcribed data, discussed the relevance of the result and drafted the manuscript together. All authors have read and approved the final manuscript.

## ETHICS APPROVAL

This study and all methods were carried out in accordance with relevant guidelines and regulations addressed in the Declaration of Helsinki, adopted by the 18th WMA General Assembly, Helsinki, Finland, June 1964. The study also follows the CODEX (2018) guidelines set by the Swedish Research Council. The Act concerning the Ethical Review of Research Involving Humans (SFS 2003:460) does not apply to this study, so Research Ethics Committee approval was not needed. Ethical considerations were observed according to the principles of information anonymity and confidentiality, voluntary participation, obtaining informed written consent, and the explanation of research goals and procedures including publication to the participants.

## CONSENT FOR PUBLICATION

All participants have given their written consent to take part in this study. Even so, the article does not contain any individual's details.

## Data Availability

The data sets generated and analysed during the current study are not publicly available due to an agreement with the participants on the confidentiality of the data but are available from the corresponding author upon reasonable request.
